# The Nucleotide-Free State of the Multidrug Resistance ABC Transporter LmrA: Sulfhydryl Cross-Linking Supports a Constant Contact, Head-to-Tail Configuration of the Nucleotide-Binding Domains

**DOI:** 10.1371/journal.pone.0131505

**Published:** 2015-06-29

**Authors:** Peter M. Jones, Anthony M. George

**Affiliations:** School of Life Sciences, University of Technology Sydney, P.O. Box 123, Broadway, NSW 2007, Australia; Russian Academy of Sciences, Institute for Biological Instrumentation, RUSSIAN FEDERATION

## Abstract

ABC transporters are integral membrane pumps that are responsible for the import or export of a diverse range of molecules across cell membranes. ABC transporters have been implicated in many phenomena of medical importance, including cystic fibrosis and multidrug resistance in humans. The molecular architecture of ABC transporters comprises two transmembrane domains and two ATP-binding cassettes, or nucleotide-binding domains (NBDs), which are highly conserved and contain motifs that are crucial to ATP binding and hydrolysis. Despite the improved clarity of recent structural, biophysical, and biochemical data, the seemingly simple process of ATP binding and hydrolysis remains controversial, with a major unresolved issue being whether the NBD protomers separate during the catalytic cycle. Here chemical cross-linking data is presented for the bacterial ABC multidrug resistance (MDR) transporter LmrA. These indicate that in the absence of nucleotide or substrate, the NBDs come into contact to a significant extent, even at 4°C, where ATPase activity is abrogated. The data are clearly not in accord with an inward-closed conformation akin to that observed in a crystal structure of *V*. *cholerae* MsbA. Rather, they suggest a head-to-tail configuration ‘sandwich’ dimer similar to that observed in crystal structures of nucleotide-bound ABC NBDs. We argue the data are more readily reconciled with the notion that the NBDs are in proximity while undergoing intra-domain motions, than with an NBD ‘Switch’ mechanism in which the NBD monomers separate in between ATP hydrolysis cycles.

## Introduction

ABC transport proteins are engaged in energy-dependent translocation of allocrites across cellular membranes [1–7]. They are found across all species and, together with a subclass of DNA repair proteins, constitute one of the largest protein families—the ABC-ATPase superfamily [8,9]. ABC transporters are involved in cellular homeostasis through the uptake of essential nutrients and cofactors and the efflux of waste. They are also involved in many other processes including extrusion of xenotoxins, hormones, lipids and liposaccharides. Several ABC transporters are implicated in significant medical problems, including multidrug resistance (MDR) in microbes and human cancers, as well as in serious inheritable diseases such as cystic fibrosis [10,11].

The conserved core architecture of ABC transporters comprises two transmembrane domains (TMDs), which form the translocation channel, and two ATP-binding cassettes which are located within the cytosol and which bind and hydrolyse ATP to energise transport. The four core domains may be expressed either separately, in pairs as half transporters or altogether on a single polypeptide [12]. Core complexes formed from separately expressed subunits, or from half transporters, may be homo- or heterodimeric, while those expressed as single polypeptides comprise pseudo-symmetrical heterodimers.

The ATP-binding cassettes or nucleotide-binding domains (NBDs) contain the Walker A and B motifs for ATP-binding [13] common to the wider family of P-loop ATPases, and the ABC signature sequence LSGGQ [14]. A number of other conserved motifs within the NBD are crucial to ATP hydrolysis and interdomain communication and include the D-, Q, and H-loops [3,15]. The ABC NBDs retain high sequence conservation, indicating a shared structure and suggesting a conserved mechanism: although structural studies have confirmed the expected structural conservation, a number of distinct mechanisms have been suggested.

Early X-ray structures of isolated homodimeric ABC NBDs confirmed our previous modelling [16] predicting they form a rotationally symmetrical dimer containing two identical ATP binding sites, whereby the LSGGQ motif of one monomer binds to ATP bound in the P-loop of the opposite monomer. This led to the suggestion the ABC NBDs function in a switch-like cycle whereby ATP binding to the separated NBD monomers induces formation of the closed sandwich dimer, whereupon ATP is hydrolysed inducing the separation of the monomers and ADP release [17]. Indeed, subsequent structures of detergent-solubilised whole ABC transporters displayed conformations in which the NBDs were completely separated, to varying extents [18]. Notably, however, despite now numerous whole ABC transporter crystal structures, both the open and closed NBD states for single ABC isoforms have proven difficult to obtain, and the closed state for a heterodimeric ABC transporter is yet to be observed.

All currently published crystal structures of whole ABC transporters are of detergent solubilised forms, and the absence of a native membrane has raised doubts whether the separated NBDs observed in whole ABC transporter structures depict natural states [19], and the physiological authenticity of the NBD-separated conformation has been questioned on the basis of enzymological data [20–23]. We have proposed an alternative “Constant Contact” scheme for the function of the ABC NBDs, in which the NBDs remain in contact in an asymmetric state, with each active site opening and closing alternately to bind and hydrolyse ATP [24]. We have shown that this scheme accords with the key data [18,25], at least as well as the NBD separation (“Switch”) model [17], which we suggested is supported most strongly by data derived from detergent-solubilised transporters [18].

In order to investigate ABC NBD separation, here we studied the homodimeric bacterial MDR LmrA from *L*. *lactis*, a well-characterised homologue of the mammalian MDR ABCB1 [26–30]. Cysteine mutagenesis and chemical cross-linking was used to probe separation between the NBDs in the nucleotide-free (apo) state. To investigate LmrA in the natural state, as distinct from detergent-containing media, we used heterologously-expressed LmrA in everted vesicles formed from *E*. *coli* whole membranes.

## Experimental Procedures

### Bacterial strains and plasmids

The source of the *lmrA* gene was *Lactococcus lactis* MG1363, generously provided by Dr Melissa Harvey, Gist-Brocades Australia Pty Ltd. *E*. *coli* strains used were DH5α (*supE44* Δ*lacU169 [*φ*80 lacZ*Δ*M15] hsdR17 recA1 endA1 gyrA96 thi-1 relA1*) and BL21-CodonPlus (DE3)-RIL (*E*. *coli* B F*- ompT hsdS(rB-mB-) dcm-* Tetr *gal* (DE3) *endA* Hte [*argU ileY leuW* Camr]; obtained from Strategene). *L*. *lactis* was propagated in MRS broth (Oxoid) or agar containing 0.5% glucose and *E*. *coli* strains in LB broth or agar. The vector pUC18 [31] was used for cloning and sequencing; and pPOWB2 [32] for function assays and cysteine oxidative cross-linking. Gene expression in pPOWB2 is regulated by the λPRPL promoter *cI*ts857 repressor system, used previously in this laboratory for the functional expression of the human *MDR1* cDNA in *E*. *coli* [33]. Vector and recombinant plasmids used in this study are listed in Table 1.

**Table 1:** Plasmids used in this study.

Plasmid	Relevant characteristic^a^
pUC18	Cloning vector
pPOWB2	Expression vector
pAMG25	lmrA in reverse order to the lacZ promoter in pUC18
pAMG26	pAMG25 but with a BclI site in the penultimate LmrA codon
pAMG28	pAMG26 but with an epitope tag inserted into the BclI site
pAMG30	lmrA in pPOWB2
pAMG31	lmrAe in pPOWB2
pAMG35	*lmrAe* A433C
pAMG36	*lmrAe* M435C
pAMG37	*lmrAe* S516C
pAMG60	*lmrAe* K388M

^a^ pUC18 and pPOWB2 were the cloning and expression vectors, respectively; all pAMG plasmids were constructed in this study.

### Cloning and expression of LmrA in *E*. *coli*

*L*. *lactis* MG1363 chromosomal DNA was isolated from an overnight MRS broth culture as described [34]. This DNA was used to amplify the *lmrA* gene using PCR and 'bookend' primers, since the sequence of only the reading frame was published [26]. The forward and reverse primers (synthesised at Sigma-Genosys, Australia) were 5'-ATGGAAAGAGGTCCACAAATGGCCAAT-3' and 5'-TTATTGACCAACAGTC AATTGTTCTGAAAC-3'. PCR cycling was performed in a Corbett Fast Thermal Sequencer FTS-320, using *Pfu* DNA polymerase (Promega). An aliquot of the reaction was run in an agarose gel and a DNA band of approximately 1.7 kb was assumed to be *lmrA*, whose ORF is 1776 bp. This fragment was ligated into the *Sma*I site of pUC18 and transformed into DH5α. Three clones, identified by asymmetric restriction analysis, all had the PCR product in the reverse orientation. One of these recombinant plasmids, designated pAMG25, was sequenced (Supamac, Australia) and shown to contain an exact copy of *lmrA* when aligned with the published sequence in ClustalW (MacVector; Oxford Molecular). DNA modifying enzymes were obtained from New England Biolabs (Australia).

### Construction of an epitope-tagged LmrA

The wild-type *lmrA* sequence in pAMG25 was modified by adding an epitope tag after the penultimate amino acid codon. The epitope tag sequence, QYPALT, was provided as oligonucleotides in all three frames with *Bam*HI adaptors; and with the specific HRP-conjugated monoclonal antibody for chemiluminescent detection of immunoblotted proteins ((I-SPY kit, AMRAD Biotech). Before doing this construction, a *Bcl*I site was introduced to provide a cloning site for the insertion of the QYPALT oligonucleotide. For this, and all subsequent constructions, we used the Strategene Quik-Change kit and *Pfu*Turbo DNA polymerase. The primer pair was: 5'-CGAGCTCGGTACCCTTATTGATCAACAGT CAATTGTTCTGAAAC-3' and 5'-AATTGACTGTTGATCAATAAGGGTACCGAGC TCGAATTCGTAATC-3'. After the PCR amplification, the change from GTT GGT CAA to GTT GAT CAA was effected, and the newly created *Bcl*I site was confirmed by nucleotide sequencing. This plasmid was designated pAMG26. The I-SPY BamHI double-stranded adaptor, 5’-GATCCTCAATACCCAGCTTTGACTCCG-3’ (with a four base overhang and rebate at the 5’ and 3’ ends, respectively, on the upper and lower strands) was ligated into the *Bcl*I site in pAMG26. The insertion was again confirmed by nucleotide sequencing and this plasmid was designated pAMG28. The last three residues in LmrA are VGQ, but the new construction produces VDPQYPALTPDQ, replacing the penultimate G residue in LmrA with the ten-residue underlined sequence, and extending the length of LmrA from 584 to 593 residues in the new version. Unless specified otherwise, all LmrA data in this study will relate to the epitope-tagged version, hereafter designated ‘LmrAe’.

Wild-type and modified *lmrA* genes were removed from pAMG25 and pAMG28, respectively, by *Eco*RI-*Bam*HI double digests and ligated directionally into the *Bam*HI-*Eco*RI sites of the expression vector pPOWB2. The *pelB* export signal sequence, just upstream of these insertions, was removed by an *Nde*I-*Bam*HI double digest and the restricted ends were blunted-ended with Klenow polymerase and ligated. These plasmids, designated pAMG30 and pAMG31, respectively, were transformed into DH5α for validation by nucleotide sequencing then transformed into BL21 (see Table 1).

### Construction of cysteine substitution mutants

**Fig 1:**
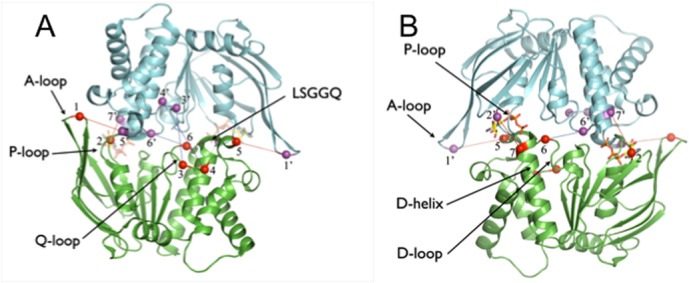
Sav1866 ATP-binding cassette closed sandwich dimer. NBD monomers are coloured green and cyan, ATP is shown in stick form and transparent to aid clarity. Cα atoms of cysteine substituted residues are shown as spheres coloured red (bottom NBD) or mauve (top NBD) and numbered, with positions within motifs in parentheses as follows: 1, S361 (A-loop); 2, P383 (P-loop); 3, A433; 4 (Q-loop), M435 (Q-loop); 5, K486 (C-motif); 6 S516 (D-loop); 7, E520 (D-helix). Numbers with a prime indicate residues on the upper monomer. Dotted lines indicate potential cross-links, and are coloured blue for the single mutants A433C, M435C and S361, and red for the double mutants S361C/K486C and P383C/E520C. (A) View from the plane of the membrane towards the face that forms the interface with the TMDs. The ‘A’, ‘P’, and ‘Q’ loops and the signature motif ‘LSGGQ’ are indicated on the structure. (B) View from the opposite side of the NBD dimer to (A), showing the underside of the NBD dimer facing the cytosol. The C-terminal α-helices have been omitted for clarity.

Native LmrA has no cysteine residues. pAMG28 was used as the template for cysteine substitutions, which were constructed by site-directed mutagenesis and PCR using Strategene's Quik-Change kit and primer sets designed to introduce the following site-specific residue changes in the LmrA NBD: A433C, M435C and S516C as mono-substitutions; and S361C/K486C and P383C/E520C as di-substitutions (Fig 1). The double mutants were constructed by a 'cut-and-paste' restriction-ligation strategy, combining pairs of mono-cysteines to produce the double mutants. To do this, use was made of a unique *Bsa*BI site within the codon for T402, which lies between each of the residue pairs of the potential di-cysteine mutants in separate clones. Complete plasmid constructs were identified in agarose gels, transformed into DH5α, purified and DNA sequenced (Supamac, Australia) to confirm the accuracy of the cysteine substitution mutants. The cysteine-substituted LmrAe constructs were subcloned directionally into the expression vector pPOWB2 as *Nco*I-*Eco*RI fragments then transformed into BL21. These clones are listed in Table 1.

### Preparation of membranes and everted vesicles

For the preparation of membranes, overnight cultures were diluted into fresh LB medium and grown with aeration at 30oC to an A600nm of 0.5. Induction was for 30 min at 42oC. Cultures were chilled then centrifuged at 4oC. Pellets were resuspended and washed twice in ice-cold Tris-buffered saline containing 100 mM EDTA and 10% glycerol. All subsequent steps were carried out at 4oC. Cell pellets were resuspended in 1/5th the original culture volume of sonication buffer [25 mM Tris-HCl (pH 7.5) containing 10 mM KCl, 10% glycerol and 1 mM PMSF]. Cell suspensions were disrupted by sonication with a 2 mm diameter probe and Branson sonifier, using 2 x 20 s pulses at half maximum power. Unbroken cells were removed by low-speed centrifugation then membranes were collected from the decanted supernatants by high-speed centrifugation. These pellets were resuspended in sonication buffer at 1/100th the original culture volume. Aliquots were snap-frozen and kept at -80oC until used. Membrane protein was estimated by a modified Lowry method [35], using bovine serum albumin as the standard.

Everted membrane vesicles were prepared essentially as described previously [36]. Briefly, cultures grown to mid-log phase in LB broth at 30oC, were induced for 30 min at 42oC, harvested and washed once in Tris-buffered saline, and resuspended at 1/100th the original culture volume in French Press buffer (100 mM Tris-HCl pH 7.5, 50 mM KCl, 2 mM DTT, 5 mM EDTA, 1 mM PMSF and 10% glycerol). After passage through a French Press at 5000 psi, unbroken cells were pelleted by high-speed centrifugation at 4oC. Vesicle pellets were collected from the supernatants by ultracentrifugation (35,000 rpm, 1 h, 4oC). These were resuspended in a small volume of French Press buffer and frozen at -80oC. Vesicle protein was estimated as before. For different samples of membranes or everted vesicles, differences in protein content were adjusted by the addition of more buffer, when the samples were thawed for ATPase assays or oxidative cross-linking.

### ATPase assays

The verapamil-stimulated ATPase activity of native and mutant LmrAs was measured as the vanadate-sensitive liberation of inorganic phosphate. Briefly, 30 μg samples of everted vesicle protein were suspended in assay buffer (50 mM Tris-HCl pH 7.5, 50 mM KCl, 2 mM DTT, 1 mM EGTA, 5 mM MgCl2 and 5 mM NaN3). Buffered suspensions of vesicles were preincubated with verapamil (600 μM) or orthovanadate (200 μM) at 37oC for 5 min. The reactions were started by the addition of MgATP (5 mM) and continued for 20 min, then stopped by the addition of an equal volume of 6% SDS. Liberated inorganic phosphate was estimated by a colorimetric ascorbic acid / molybdate assay [37]. A standard curve for inorganic phosphate was prepared using Na2HPO4. ATPase activity was obtained by subtracting the background or basal activity in the presence of orthovanadate from the verapamil-stimulated activity. Dithiothreitol was used in the reactions to prevent cysteine cross-linking that might moderate ATPase activity. All assays were performed twice in triplicate. The control for endogenous ATPase activity was LmrA in which the Walker A lysine was substituted by methionine, which results in the abrogation of ATPase activity [28]. This K388M LmrA mutant was constructed by PCR site-directed substitution in the same way as the cysteine mutants described above. The forward and reverse primers were: 5'-GTCCTTCTGGTGGTG GTATGTCAACCATCTTCTCACTTTTAG-3' and 5'-TTCTAAAAGTGAGAAGATGGT TGACATACCACCACCAGAAGG-3'.

### Sulfhydryl cross-linking in membranes, immunoblotting and detection

Oxidative cross-linking of cysteines was carried out in the presence of Cu(phenanthroline)32+. Each cross-linking reaction contained 10 μg of membrane protein, 5 mM ATP (when used), 3 mM CuSO4 and 9 mM 1,10-phenanthroline, in a total volume of 50 μl. Identical reaction mixtures were set up with or without 5 mM ATP, oxidative copper reagent, or 5 mM DTT. Reactions were allowed to proceed for 60 min at 4o or 21oC, before being quenched by the addition of an equal volume of stop buffer [38]. These sample mixtures were used immediately or were stored at -20oC until needed. Samples in stop buffer were heated at 95oC for 3 min then were subjected to electrophoresis in 7.5% SDS-polyacrylamide Laemmli gels; electroblotted onto nitrocellulose sheets (Amersham Pharmacia Biotech) at 4°C in transfer buffer (25 mM Tris-HCl pH 7.5, 190 mM glycine, 20% (v/v) methanol); probed with the I-Spy10 HRP-conjugated monoclonal antibody against the QYPALT peptide in LmrA mutants, using the I-Spy protocol (AMRAD Biotech); and detected by enhanced chemiluminescence (Amersham ECL system) on X-ray film (Biomax ML, Kodak).

When samples of all cross-linking reactions were treated with DTT and then subjected to electrophoresis and immunoblotting, only single bands were observed in gels at the molecular size of the LmrA monomer (not shown), which is as expected when the disulfide bond is reduced in the presence of DTT [39,40].

## Results and Discussion

### Cloning of LmrA

The *lmrA* gene from chromosomal DNA from the *L*. *lactis* MG1363 strain was subcloned into the low copy number vector pPOWB2, with a tight repressor/promoter system. *LmrA* was then modified by introducing an epitope tag sequence into the penultimate glycine codon. The size and location of LmrA was checked in SDS-polyacrylamide gel, run with duplicate lanes of membrane proteins from uninduced and induced cultures of BL21 (pAMG30; wild-type LmrA) and BL21 (pAMG31; LmrAe). One half of the gel was stained with Coomassie Blue and the other half was immunoblotted and developed with the I-SPY monoclonal antibody. Protein bands of approximately the same molecular size were identified in the induced lanes only of the stained gel; and a single new band of about 66 kDa was detected in LmrAe lane only of the antibody-treated blot of the duplicate half of the gel (not shown). The LmrAe clone was used in the cysteine substitution experiments. The *lmrA* gene has been cloned in *E*. *coli* previously [26], but that study was limited to studying the MDR property of LmrA as a model for other MDR ABC transporters.

### Construction of cysteine mutants

**Fig 2:**
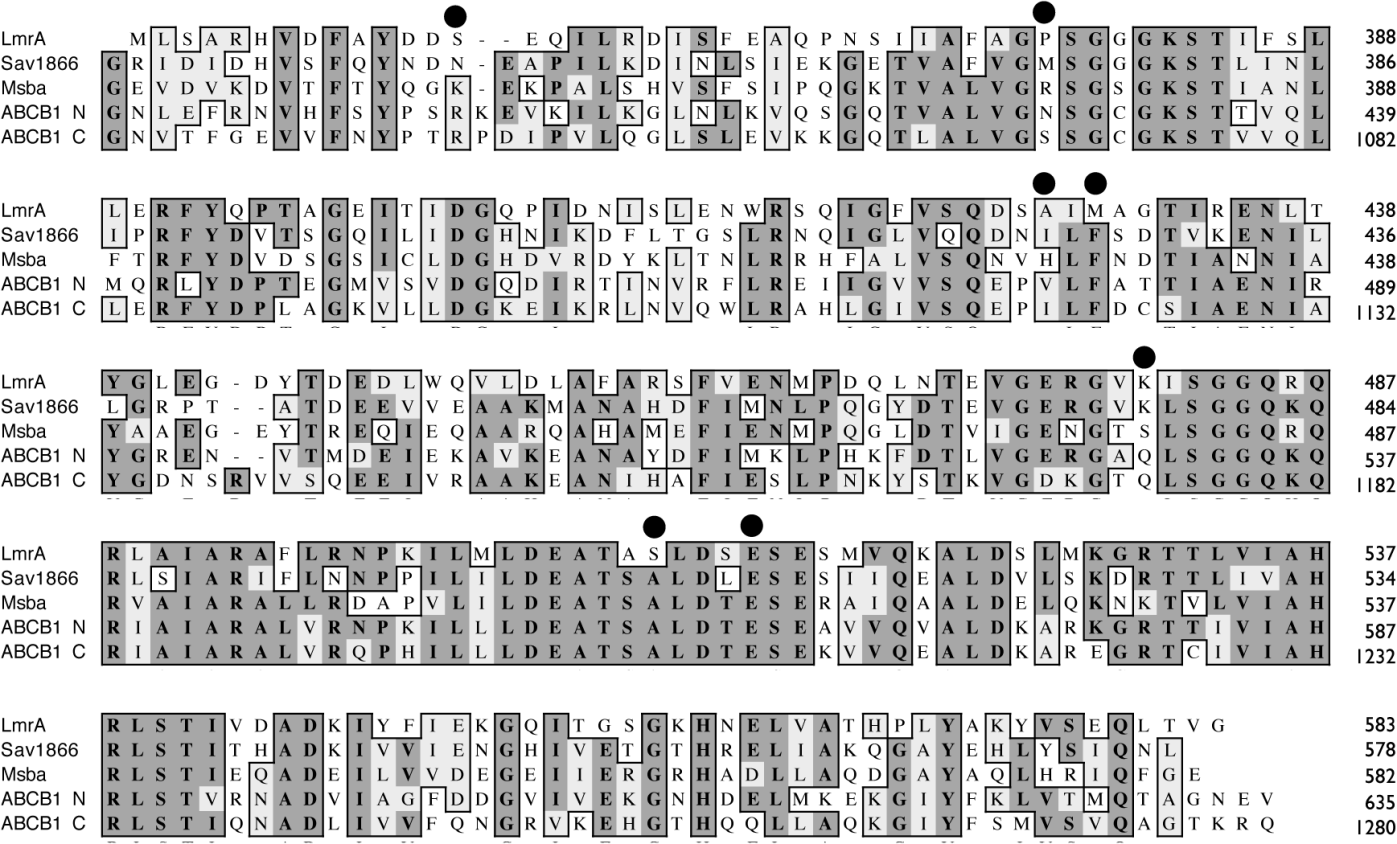
ClustalW alignment of ABC transporter NBDs. The alignment and was used to identify equivalent residues in Sav1866, MsbA, and ABCB1 (N- and C-terminal NBDs) to those of LmrA selected for cysteine mutagenesis and cross-linking, identified with black dots. Residues are numbered according to the primary sequences of the transporters. Fully conserved residues are shaded dark grey, with similar residues in light grey. The UniProtKB database identifiers for the primary sequences were: LmrA (*L*. *lactis*, Q9CHL8); Sav1866 (*S*. *aureus*, Q99T13); MsbA (*E*. *coli*, P60752); ABCB1 (*H*. *sapiens*, P08183).

Target residues for cysteine substitution mutagenesis were determined on the basis of proximity across the interface of the closed ABC NBD dimer. Although a crystal structure of the LmrA NBD dimer is not available, a structure of the LmrA NBD monomer is, and this was used together with a structure of the LmrA homologue and bacterial ABC MDR, Sav1866 (2HYD), to estimate intermonomer distances (Fig 1). The MDR activity of LmrA and Sav1866, as well as of the bacterial lipid A transporter MsbA and the mammalian MDR ABCB1, have been well-characterised. Consistent with the sequence homology between these proteins (Fig 2), they display overlapping substrate specificity profiles [27,41,42]. This suggests Sav1866 is a suitable structural template for the LmrA NBD dimer, and that inferences drawn regarding one or more members of this group of ABC MDRs are likely to have relevance for the others.

An automatic CLUSTAL sequence alignment was made of LmrA with Sav1866, *V*. *cholerae* MsbA and the N- and C-terminal NBDs of human ABCB1 (Fig 2). The sequence alignment between LmrA and Sav1866 was verified by structural fitting using the crystal structure of the LmrA NBD monomer (1MV5) with Sav1866, and distances across the closed LmrA NBD dimer interface for specific residue pairs then estimated from the distances between homologous residues in the Sav1866 structure (Fig 1).

Both single and double mutants were created, at positions predicted to be within, or close to cross-linking distance in the closed NBD dimer. Since LmrA is a homodimer, each single mutant is therefore duplicated in the homodimeric transport complex and capable of forming one sulfhydryl cross-link, while for the double mutants two cross-links potentially can be formed. For the single mutants, residues targeted for substitution were: A433, M435, and S516. Residues A433 and M435 are on the Q-loop, 3 and 5 residues downstream of the conserved glutamine. A433 is equivalent to A85 in the NBD of the *S*. *typhimurium* maltose permease, MalK; this position has been used previously to assess the separation of MalK subunits using chemical cross-linking [39,43] and EPR [44]. S516 is on the D-loop, at a position equivalent to that immediately C-terminal to residue G174 in the Archaea ABC NBD MJ0796, at which point a tryptophan substitution was used to monitor proximity of the isolated NBD [45].

The double mutants contained the pairs S361C/K486C and P383C/E520C; single mutants for each of these four positions were also created as controls. S361 is on the A-loop [46], while K486 is the residue immediately preceding the LSGGQ motif. P383 is part of the P-loop, being the second residue of the Walker A motif, and E520 is near the N-terminus of the α-helix immediately downstream of the D-loop [15,47], two residues past the conserved aspartate.

### Measurement of verapamil-stimulated ATPase activities

**Fig 3:**
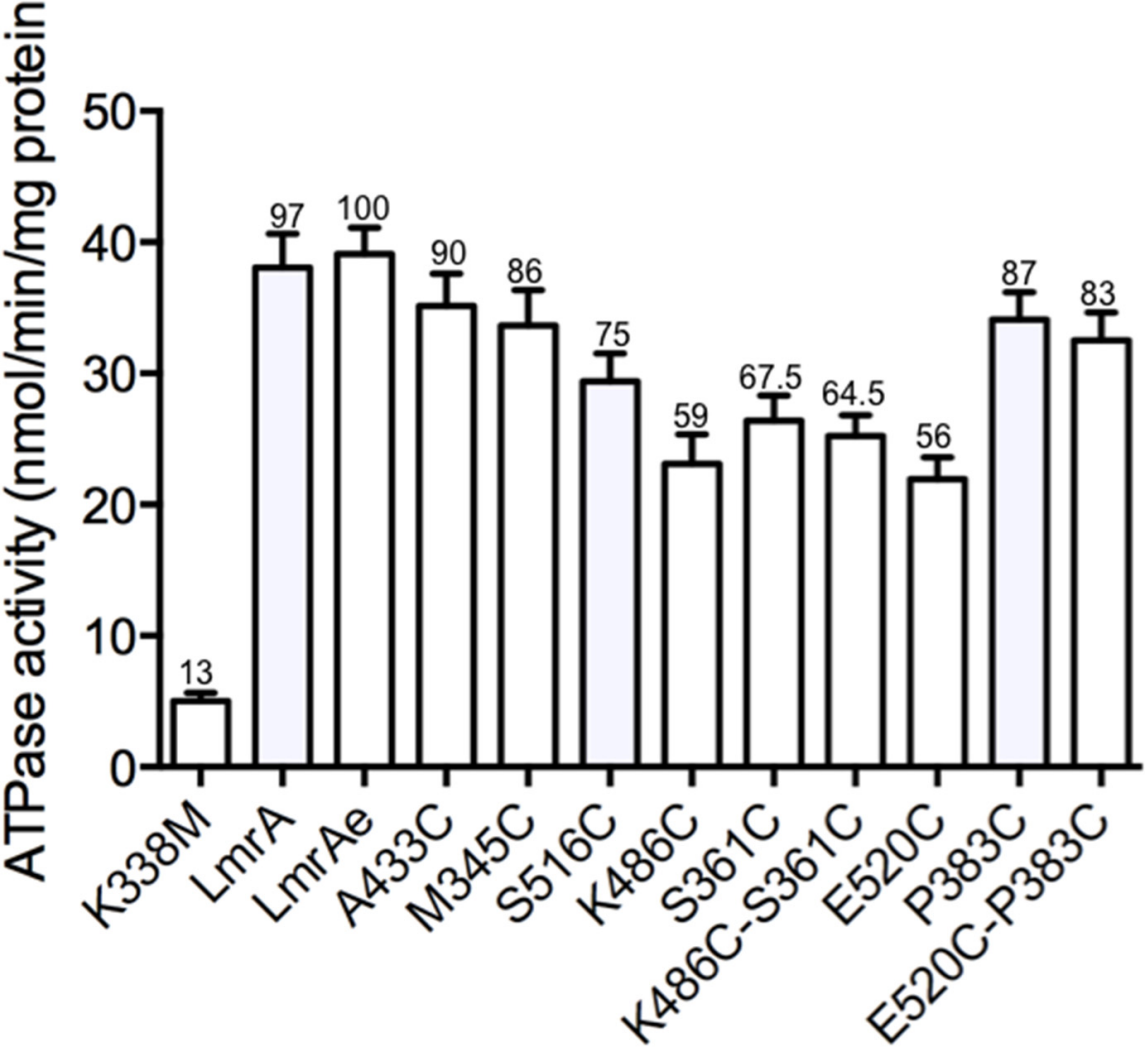
Verapamil-stimulated ATPase activities of LmrA cysteine substitution mutants. ATPase activities were determined as the differences between verapamil-stimulated and vanadate-sensitive release of inorganic phosphate as described in ‘Experimental Procedures.’ The numbers at the top of each bar are the percentage activities relative to native LmrA (100%) with the absolute ATPase activities shown on the y-axis. All of the mutants were derived from LmrAe. The K388M mutant is the control for basal ATPase activity. All other mutants were cysteine substitutions of the primary sequence residues indicated on the x-axis. Each bar represents the average of three measurements.

The suitability of the mutants for sulfhydryl cross-linking reactions was assessed in everted membrane vesicles by ATPase activity assays. These data are summarised in Fig 3. Firstly, there was no difference between the ATPase activities of native LmrA (97%) and modified LmrAe (100%), indicating that the attachment of the epitope tag had negligible effect on hydrolytic activity. All of the single and double cysteine mutants exhibited 57–87% of the activity of LmrAe. The lowest activities relative to LmrAe were exhibited by the single mutants E520C (56%) and K486C (59%), but these activities rose to 83% and 64.5% in the respective double mutants, P383C/E520C and S361C/K486C. This range of ATPase activities typically similar to those of many cysteine mutants generated in other ABC transporters [48–51]. The background activity of a Walker A-impaired mutant (K388M) was only 13% of the activity relative to LmrAe, indicating that changing the Walker A lysine residue to methionine abrogated most of the ATPase activity, consistent with the previous finding for this K388M mutant in LmrA [28].

### Oxidative cysteine cross-linking between the NBD subunits of the LmrA dimer

In this study, cysteine mutagenesis and sulfhydryl cross-linking in the *L*. *lactis* MDR ABC transporter LmrA was used to probe the separation of the NBD monomers in the nucleotide-free state. LmrA was chosen because it has been functionally expressed in *E*. *coli* [26], and because it has the advantage of containing no native cysteine residues.

**Fig 4:**
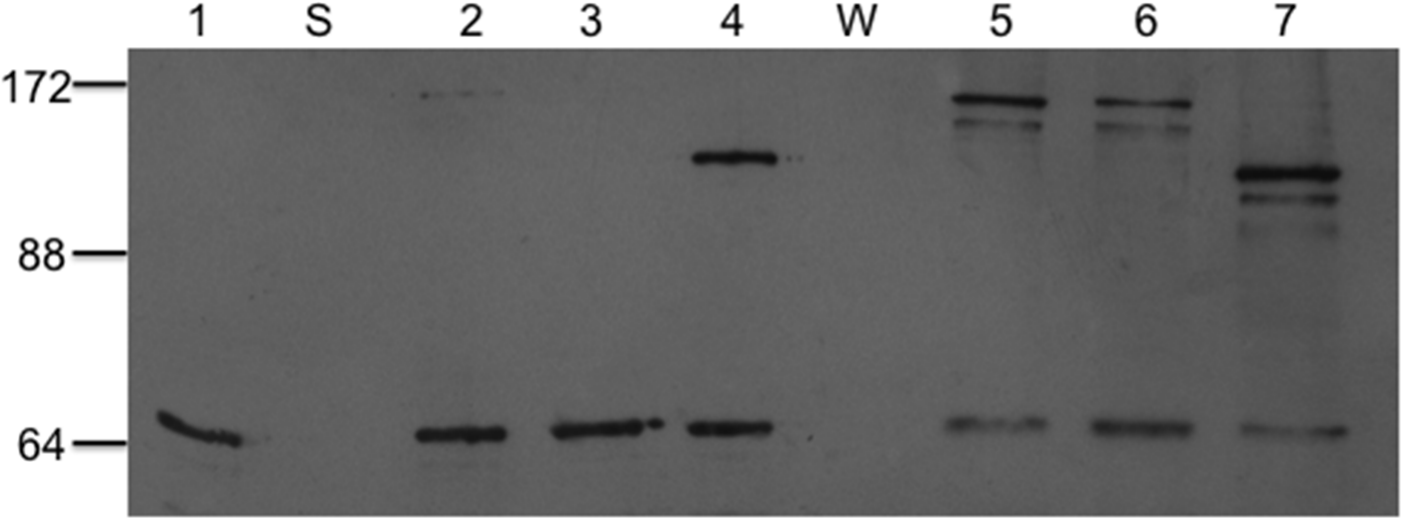
Sulfhydryl cross-linking of single LmrA cysteine substitution mutants. Immunoblotted SDS-polyacrylamide 7.5% gels with 10 μg of membrane protein run in each lane. Lane 1 contains LmrA wild-type (cysteine-less); lanes 2, 3 and 4 contain mutants A433C, M435C and S516C, respectively, cross-linked at 4°C; and lanes 5, 6, and 7, the same mutants cross-linked at 21°C. The different positions for the dimers is due to different cross-linked positions giving rise to dissimilar configurations of the linearised cross-linked dimers and hence alterations in their mobilities. Molecular size positions were run in the lane marked ‘S’. The LmrAe monomer in the lane marked ‘W’ has an apparent molecular mass of 66 kDa.

For the single mutants A433C, M435C, and S516C at 4°C and 21°C, only S516C showed significant cross-linking at both temperatures, while A433C and M435C showed some cross-linked product only at 21°C, with A433C showing greater cross-linking than M435C (Fig 4). The results suggest that at the higher temperature increased thermal motion brings more distant residues into cross-linking range more frequently. The observed pattern of cross-linking is in accord with the relative distances between the positions predicted in the closed NBD dimer (Table 2). These data are thus consistent with the view that when the LmrA NBDs interact, they do so in the manner at least approximating the sandwich dimer configuration, and that the cross-linking at 4oC more clearly defines inter-residue distances, only allowing residues under 14Å to produce clear cross-linked product. Fig 4 (lane 1) depicts the cysteineless LmrAe subjected to the same cross-linking reaction as the mutants. As expected, there was only a single monomer band at approximately 66 kDa.

**Table 2:** Predicted distances (Å) between Cα atoms of the same or different residue pairs in NBD dimers of the half transporters LmrA, Sav1866, and MsbA.

LmrA	Sav1866 (2HYD)		MsbA V. cholerae (3B5X)	
Residue pair	Residue pair	Predicted distance (Å)	Residue pair	Predicted distance (Å)
A433 / A433	I425 / I425	14.1	H427 / H427	38.4
M435 / M435	F427 / F427	20.1	F429 / F429	45.8
S516 / S516	A507 / A507	9.3	A510 / A510	40.3
S361 / K486	- / K477 ^a^	12.1	K354 / S480 ^b^	22.45
S361 / S361	- /- ^a^	50.8	K354 / K354 ^b^	28.3
K486 / K486	K477 / K477	26.7	S480 / S480	60.6
P383 / E520	M375 / E511	7.0	R377 / E514	38.4
P383 / P383	M375 / M375	27.6	R377 / R377	12.4
E520 / E520	E511 / E511	23.3	E514 / E514	64.5

The LmrA residue pairs used in this cross-linking study are identified in the first column; pairs for which clear cross-linked products were observed are shown in bold font. The equivalent pairings for Sav1866 and MsbA and the predicted distances between the residue pairings are given in columns 2–5. The indicated residue numbers correspond to the primary sequences identified in the alignment (Fig 1).

^a^ Distances for LmrA S361 in the sandwich dimer were estimated by structural alignment of the LmrA NBD structure (PDB: 1MV5) and the Sav1866 structure (2HYD), using Cα atoms of the NBD core subdomains (LmrA residues 342–352, 358–406, 500–505, 530–536, and 545–561) and measuring from LmrA S361.

^b^ Distances for LmrA S361 in the inward-facing closed conformation were estimated by measuring distances from K354 in *V*. *cholerae* MsbA (3B5X).

The results for the S516C single mutant at 4°C and 21°C suggest that, given that sulfhydryl cross-linking will not proceed to completion even for optimally placed residues, a substantial proportion of the NBDs are either in contact, or come into contact over the 60-minute incubation. From the perspective of the NBD separation model, the clear increase in cross-linking between 4°C and 21°C for the A433C, M435C, and S516C single mutants indicates that in the absence of nucleotides, transition from the open to the closed state can be thermally induced. Moreover, this transition occurs to a substantial extent even at 4°C. Given that only a fraction of closure events will result in cross-linking, that ATPase activity is abrogated at this temperature, and that ATP is not present to drive this “power stroke”, estimated to have an activation energy around 100kJ/mol for basal ATPase activity in ABCB1 [52], it appears unclear how the extent of cross-linking observed here for nucleotide-free LmrA at 4°C can be reconciled with the NBD separation or Switch Model [17].

From the perspective of the Constant Contact Model [24], it should be noted that at least one nucleotide is proposed to be bound at all times, making the nucleotide-free situation unnatural or non-physiologic and its conformation uncertain. Nevertheless, NMR data for LmrA in the nucleotide-free state [30] showed that, while the TMDs are relatively static, the NBDs are highly mobile. We have previously suggested this mobility in LmrA can be attributed to motions of NBD subdomains, on the basis of MD simulations of Sav1866 [53]. Thus, in this view, the NBDs are in proximity in a head-to-tail orientation, albeit with high mobility of their principal subdomains, and thus are able to form cross-links, with thermal motions allowing increased cross-linking by bringing residues into contact more frequently at higher temperatures.

**Fig 5:**
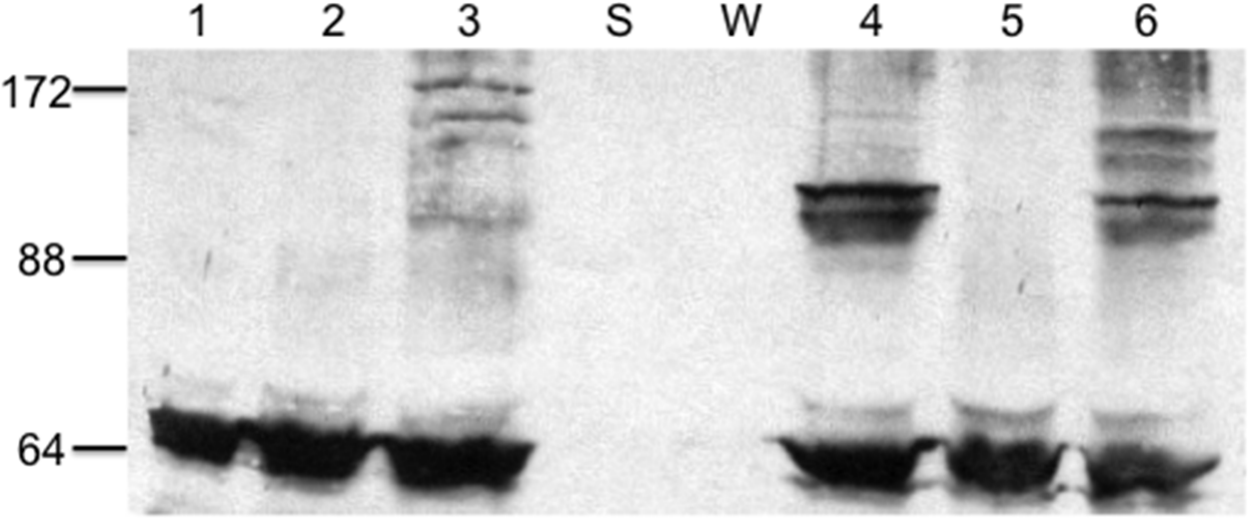
Sulfhydryl cross-linking of double LmrA cysteine substitution mutants. Immunoblotted SDS-polyacrylamide 7.5% gels with 10 μg of membrane protein run in each lane. Lanes 1, 2, and 3 contain S361C, K486C single mutants and the S361C / K486C double mutant, and lanes 4, 5, and 6 contain E520C, P383C single mutants and the P383C / E520C double mutant, respectively. Samples were cross-linked at 4°C in the presence of oxidative copper and ATP. Molecular size positions were run in the lane marked ‘S’. The LmrAe monomer in the lane marked ‘W’ has an apparent molecular mass of 66 kDa.

Cross-linking reactions for the double mutants S361C/K486C and P383C/E520C were performed at 4°C. In Fig 5, there was no appreciable cross-linked product for the single S361C and K486C mutants, but substantial cross-linking occurred in the S361C/K486C double mutant. This pattern of cross-linking supports the head-to-tail NBD dimer configuration, inasmuch as the single mutants, for which the substituted positions are predicted to be clearly beyond cross-linking distance (>25Å, Table 2), do not show cross-linked product, while the double mutant, with predicted Cα-Cα distances of about 12Å, does form cross-links.

Notably, the Cα-Cα distance for the single mutant A433C is predicted to be at a similar distance (14Å) in the sandwich dimer, but does not show appreciable cross-linked product at 4°C. However, Q-loop A433C is buried against coupling helix 2, which occupies the space between the two A433Cs (Fig 2). Whereas S361C is situated on the A-loop, which has been consistently observed to be relatively mobile in MD simulation analysis of a range of ABC NBDs: HisP [54,55]; MsbA [56]; MJ0796 [57–59]; BtuD [60]; MalK [61]; Sav1866 [62]; HlyB [63]; CFTR [64]; ABCB1 [65]. Moreover, the Cα-Cα distance for LmrA S361C/K486C is an estimate only, since the Sav1866 A-loop is longer by one residue (Fig 2), and so the actual S361C/K486C Cα-Cα distance in LmrA may be shorter than 12Å. Finally, in contrast to A433C, no peptide is predicted to intervene between S361C and K486C (Fig 2). Thus, the presence of cross-linked product observed for double mutant S361C/K486C but its absence in the single mutant A433C, despite their similar predicted separations, may be reconciled with a sandwich dimer configuration.

For the single P383C mutant no cross-linking was detected, while for the E520C single mutant, cross-linked product was clearly detected, and a substantial amount of cross-linking occurred for the P383C/E520C double mutant, which was notably more than that of the S361C/K486C pair (Fig 5). While cross-linking in the P383C/E520C double mutant and its absence in the P383C single mutant are consistent with the sandwich dimer configuration, the presence of cross-linked product in the E520C single mutant, where the residues are predicted to be over 23Å apart, is not.

Since the E520C single mutant result alone appears markedly at odds with a sandwich dimer configuration, it might be suggested that the observed cross-linked product was due to cross-links formed between separate LmrA complexes and/or with other membrane proteins. However, if this were the case, why did S361C, K486C, and P383C single mutants not similarly form detectable cross-linked products? Since E520C does not appear significantly more exposed than S361C, K486C or P383C, to account for this, it appears more likely that single mutant E520C forms intra-complex cross-links.

The data for the double mutants together with their associated single mutants further indicate that the NBDs come into contact in the absence of substrate and nucleotide at 4°C. As discussed above, this appears difficult to reconcile with the NBD Switch Model. Of relevance to this, two recent studies of homologous ABC MDRs also found the NBDs were in proximity in the nucleotide-free state [66,67]. Moeller et al., (2015) [67] suggested that in the nucleotide-free state, a subset of transporters adopt an “inward-closed” conformation observed in an X-ray structure of the nucleotide-free *V*. *cholerae* MsbA isoform [68]. In this structure, the NBDs are close enough to be in direct contact, but are separated overall relative to the sandwich dimer, and shifted laterally, approximately along the plane of symmetry dividing them, bringing the P-loops into closer proximity. This idea is also supported by the analysis by Sim et al., (2013) [66] who found that native cysteines in the P-loops came to within 20Å in the nucleotide-free state, more than 10Å closer than their separation predicted in the closed NBD dimer.

However, as shown in Table 2, the inward-facing closed state observed for *V*. *cholerae* MsbA does not accord with the pattern of cross-linking reported here for LmrA. In particular, it is noteworthy that, based on this structure and homology between MsbA and LmrA (Figs 1 and 2), residues S516C/S516C’ are predicted to be over 40Å apart while residues E520C/E520C’ are around 65Å apart, yet in the present study, single mutants at both positions show substantial cross-linking. Also, according to the *V*. *cholerae* MsbA structure, the LmrA pair P383C/P383’ is predicted to have a Cα-Cα distance of 12.4Å, and here do not cross-link (Fig 5, lane 5), while S361C and K486C are predicted to be over 20Å apart, but here do cross-link (Fig 5, lane 3).

From the perspective of the constant contact model of ABC NBD interaction, the data for the double mutants S361C/K486C and P383C/E520C can largely be explained as described above: The NBDs are in proximity in a head-to-tail configuration while undergoing dynamic motions of their core subdomains. The only result that requires further explanation is the cross-linked product observed for the E520C single mutant, who’s Cαs are predicted to be 23Å apart in the sandwich dimer. Previously we found in MD simulations of Sav1866 that the α-helix immediately following the D-loop (D-helix) is mobile, being able to pivot about a hinge at its C-terminus [15]. In addition, EPR experiments using the maltose permease have indicated the D-helix is mobile relative to the helical subdomain [69]. Thus, given that E520C is situated at the N-terminus of the D-helix, we suggest that mobility of the D-helix, allied to the mobility of the core subdomain, allows the two E520C to come into contact.

## Conclusion

The data presented here for the ABC MDR transporter LmrA indicate that in the absence of nucleotide or substrate, the NBDs come into contact sufficient to allow extensive cross-linking, even at a temperature where ATPase activity is abrogated. From the perspective of the Switch mechanism, it is not clear how the contact between NBDs detected here can be reconciled with the idea that ATP is required to induce the dimerisation of the NBDs. Also, from this perspective, the contact between the N-termini of the D-helices observed here (E520C/E520C’) appears unexplained.

The pattern of cross-linking obtained in this study clearly does not support an inward closed conformation akin to that observed in the crystal structure of *V*. *cholerae* MsbA. Rather, the data suggest a head-to-tail configuration similar to that observed in crystal structures of nucleotide-bound ABC NBDs. The results are consistent with the idea that the NBDs are in proximity, while undergoing intradomain motions, as suggested in our Constant Contact Model. Indeed, it appears difficult otherwise to reconcile the dynamics of nucleotide-free LmrA observed in NMR studies, in which the NBDs were found to be highly mobile while the TMDs were relative static. Notably, this dynamism in the context of a head-to-tail NBD dimer configuration provides an explanation for the observed cross-linking of E520C/E520C’ which is not explained by the inward-closed conformation seen in MsbA [67], or by a rigid switch motion of the NBDs between open and closed states.

We have argued previously that the NBD separated conformation is an artefact caused by the unnatural absence of nucleotide and/or the experimental conditions. Notably, in a recent EM study [67], the premise of the NBD separated state as a functional intermediate, as opposed to an artefact, required the attribution of distinctly different mechanisms to the homologues ABCB1 and MsbA in the interpretation of the data, despite these MDR exporter ABC proteins clearly being closely related. We believe that, notwithstanding burgeoning suggestions of distinct mechanisms among ABC transporters, such ideas are at odds with the conserved nature of the ABC NBDs.
